# Clinical likelihood ratios and balanced accuracy for 44 in silico tools against multiple large-scale functional assays of cancer susceptibility genes

**DOI:** 10.1038/s41436-021-01265-z

**Published:** 2021-07-06

**Authors:** C. Cubuk, A. Garrett, S. Choi, L. King, C. Loveday, B. Torr, G. J. Burghel, M. Durkie, A. Callaway, R. Robinson, J. Drummond, I. Berry, A. Wallace, D. Eccles, M. Tischkowitz, N. Whiffin, J. S. Ware, H. Hanson, C. Turnbull, CanVIG-UK

**Affiliations:** 1 Division of Genetics and Epidemiology, Institute of Cancer Research, Sutton, UK; 2 Manchester Centre for Genomic Medicine and NW Laboratory Genetics Hub, Manchester University NHS Foundation Trust, Manchester, UK; 3 Sheffield Diagnostic Genetics Service, North East and Yorkshire Genomic Laboratory Hub, Sheffield Children’s NHS Foundation Trust, Sheffield, UK; 4 Wessex Regional Genetics Laboratory, Salisbury Hospital NHS Foundation Trust, Salisbury, UK; 5 Human Genetics and Genomic Medicine, Faculty of Medicine, University of Southampton, Southampton, UK; 6 Yorkshire and North East Genomic Laboratory Hub, Leeds Teaching Hospitals NHS Trust, Leeds, UK; 7 East Genomic Laboratory Hub, Cambridge University Hospitals Genomic Laboratory, Cambridge University Hospitals, Cambridge, UK; 8 Cancer Sciences, Faculty of Medicine, University of Southampton, Southampton, UK; 9 Department of Medical Genetics, National Institute for Health, Research Cambridge Biomedical Research Centre, University of Cambridge, Cambridge, UK; 10 Wellcome Centre for Human Genetics, Oxford, UK; 11 National Heart and Lung Institute & MRC London Institute of Medical Sciences, Imperial College London, London, UK; 12 Royal Brompton & Harefield Hospitals, London, UK; 13 Department of Clinical Genetics, St. George’s University Hospitals NHS Foundation Trust, London, UK; 14 Cancer Genetics Unit, Royal Marsden NHS Foundation Trust, London, UK

## Abstract

**Purpose:**

Where multiple in silico tools are concordant, the American College of Medical Genetics and Genomics/Association for Molecular Pathology (ACMG/AMP) framework affords supporting evidence toward pathogenicity or benignity, equivalent to a likelihood ratio of ~2. However, limited availability of “clinical truth sets” and prior use in tool training limits their utility for evaluation of tool performance.

**Methods:**

We created a truth set of 9,436 missense variants classified as deleterious or tolerated in clinically validated high-throughput functional assays for *BRCA1*, *BRCA2*, *MSH2*, *PTEN*, and *TP53* to evaluate predictive performance for 44 recommended/commonly used in silico tools.

**Results:**

Over two-thirds of the tool–threshold combinations examined had specificity of <50%, thus substantially overcalling deleteriousness. REVEL scores of 0.8–1.0 had a Positive Likelihood Ratio (PLR) of 6.74 (5.24–8.82) compared to scores <0.7 and scores of 0–0.4 had a Negative Likelihood Ratio (NLR) of 34.3 (31.5–37.3) compared to scores of >0.7. For Meta-SNP, the equivalent PLR = 42.9 (14.4–406) and NLR = 19.4 (15.6–24.9).

**Conclusion:**

Against these clinically validated “functional truth sets," there was wide variation in the predictive performance of commonly used in silico tools. Overall, REVEL and Meta-SNP had best balanced accuracy and might potentially be used at stronger evidence weighting than current ACMG/AMP prescription, in particular for predictions of benignity.

## INTRODUCTION

### Variant interpretation

For more than three decades, sequence analysis of constitutional DNA has informed diagnosis and prediction of human Mendelian diseases. Robust identification of the causative pathogenic variant enables accurate prediction of the clinical course of disease and implementation of measures for prevention and early detection. Through technological advances, clinical genome sequencing is now routine, typically revealing in comparison to a reference genome in excess of 4 million variants in the average human [1]. Through concerted efforts within the clinical community to reduce erroneous assignation of variants as pathogenic, common frameworks for variant interpretation have been evolved, such as that of the American College of Medical Genetics and Genomics/Association for Molecular Pathology (ACMG/AMP). Within this system, points are tallied up from quasi-orthogonal lines of evidence, such as clinical case series, segregation data, phenotypic specificity, and laboratory assays [2].

### Emergence of in silico tools

However, rare missense variants are frequently identified on clinical genetic testing that have not previously been reported or for which existing clinical and laboratory data are sparse. In these scenarios, evaluation of variant pathogenicity/benignity is largely reliant on predicted alteration of protein function using features such as the following:

#### Homology in sequence alignment between divergent species

Orthologs are gene sequences derived from the same ancestral gene present in two species’ last common ancestor. Where an amino acid is highly conserved across multiple orthologs, this indicates that a change in that amino acid will be of deleterious consequence for protein function.

#### Physiochemical differences between amino acids

Amino acids are characterized by their composition, polarity, and molecular volume. A large physiochemical distance for a substitution would be predicted to have a greater impact on protein function [3].

#### Disruption to 3D protein structure

Amino acid substitutions are more likely to be deleterious if they alter tertiary protein structure, including folding, bonds, and binding site shapes [4]. While protein structure can be visualized directly using X-ray crystallography and nuclear magnetic resonance (NMR), most predictions are largely based on modeling.

Over the last 20 years, computational biologists have developed a number of in silico prediction algorithms or tools variously leveraging these features. In addition to knowledge of biological principles, in silico tools may be trained against a truth set in which impact of variants on protein function is already quantified [5]. The performance of the tool may then be validated or tested against other independent truth sets. Early training truth sets typically included prokaryote assays of broad cell function: given the divergence between humans and yeast and the complex cellular functions of many human disease-associated genes, such predictions are likely to relate only loosely to clinical pathogenicity of human disease genes [6]. However, more recently, large-scale databases of clinical and neutral population variation have been made publicly available, such as ClinVar, Human Gene Mutation Database (HGMD), SwissProt, and ExAC/gnomAD [7–10].

There has been a surge in release of new tools and metapredictors (tool combinations) largely trained on these same data sets. With multiple elements incorporated into sophisticated machine learning algorithms, training on restricted data sets has potential to result in overfitting, that is, recognition of features present within the training data set due to random variation, rather than those that are useful for prediction in new data sets [11–13]. In the case of metapredictors, this may result in the constituent tools appearing to perform better and therefore being allocated excess weight within the overall algorithm [14]. The field has been further confused by intertool comparisons using the same data sets upon which they were trained [11].

In addition, some clinical databases may offer less reliable variant classifications: earlier instances of HGMD, for example, largely assigned as pathogenic any variant detected in an individual with the relevant phenotype, while only more recently has ClinVar curation tackled erroneous community submissions [15, 16]. Such databases may also be biased toward variants with features that are more easily detectable by prediction software, as conclusive clinical classifications are less frequently established for more “challenging” variants [11]. Furthermore, differences in tools performance have been observed across different populations and between different variant types (gain of function vs. loss of function and pathogenic vs. benign) [17–19].

We summarize the data inputs, methodologies, and testing/training data underpinning 45 in silico tools widely used in clinical practice and/or available from amalgamation sites [20, 21] (Table 1, Supplementary Table 1).

**Table 1:** Forty-five in silico tools evaluated including components, training and test data sets.

Tool name		Data output		Components of tool					Training data set(s)					Test (validation) data set(s)				
		Categorical	Continuous	Evolutionary conservation	Protein features	Amino acid features	Nucleotide features	Clinical/population variation	Clinical classifications: ClinVar	Clinical classifications: HGMD	Clinical classifications: other	Nonclinical: human variation	Nonclinical: functional studies	Clinical classifications: ClinVar	Clinical classifications: HGMD	Clinical classifications: other	Nonclinical: human variation	Nonclinical: functional studies
Align-GVGD		✓	✗	✓		✓					✓	✓				✓	✓	✓
BayesDEL		✗	✓	✓	✓	✓	✓	✓	✓		✓	✓				✓	✓	
CADD		✗	✓	✓	✓	✓	✓					✓		✓	✓	✓	✓	✓
CHASM		✗	✓	✓	✓	✓		✓				✓				✓		
ClinPred		✗	✓	✓	✓	✓	✓	✓	✓					✓			✓	✓
Condel		✗	✓	✓	✓	✓						✓					✓	✓
DANN		✗	✓	✓	✓	✓	✓					✓		✓			✓	
Eigen		✗	✓	✓	✓	✓	✓					✓		✓		✓	✓	
FATHMM		✗	✓	✓	✓					✓		✓				✓	✓	
FATHMM-MKL		✗	✓	✓	✓		✓			✓		✓			✓		✓	
fitCons		✗	✓	✓	✓		✓						✓					
GAVIN		✓	✗	✓	✓	✓	✓	✓	✓			✓		✓	✓	✓	✓	
GenoCanyon		✗	✓	✓	✓		✓					✓		✓				
GERP++		✗	✓	✓								✓						
Grantham score		✗	✓	✓		✓												
LRT		✗	✓	✓								✓						
M-CAP		✗	✓	✓	✓	✓	✓	✓		✓		✓			✓		✓	
Meta-SNP		✗	✓	✓	✓	✓						✓					✓	
MetaLR		✗	✓	✓	✓	✓	✓	✓				✓				✓	✓	
MetaSVM		✗	✓		✓								✓				✓	✓
MLP		✗	✓	✓	✓	✓	✓	✓				✓				✓	✓	
MSC		✓	✗	–	–	–	–	–	✓	✓		✓						✓
MutationAssessor		✓	✓	✓								✓					✓	✓
MutationTaster2		✓	✗	✓	✓		✓	✓		✓		✓			✓		✓	
MutPred		✗	✓	✓	✓					✓		✓			✓		✓	✓
MVP		✗	✓	✓	✓	✓	✓	✓	✓	✓		✓				✓	✓	
PANTHER		✗	✓	✓	✓							✓						
PhastCons	PhastCons100way	✗	✓	✓								✓						
	PhastCons20way	✗	✓	✓								✓						
PhD-SNPg		✓	✓	✓	✓				✓					✓				
PhyloP	PhyloP100 verebrate	✗	✓	✓								✓						
	PhyloP20 mammalian	✗	✓	✓								✓						
Pmut		✓	✓	✓	✓	✓						✓		✓			✓	
PolyPhen-2	HumDiv	✗	✓	✓	✓	✓						✓					✓	
	HumVar	✗	✓	✓	✓	✓						✓					✓	
PON-P2		✓	✓	✓	✓	✓						✓					✓	
PredictSNP		✓	✓	✓	✓	✓				✓		✓					✓	✓
primateAI		✗	✓	✓	✓	✓		✓	✓			✓				✓		
PROVEAN		✗	✓	✓		✓	✓					✓			✓		✓	✓
REVEL		✗	✓	✓	✓	✓		✓		✓		✓		✓	✓		✓	
rfPred		✗	✓	✓	✓	✓	✓	✓			✓	✓		✓			✓	
SIFT		✗	✓	✓									✓				✓	
SiPhy		✗	✓	✓								✓					✓	
SNAP2		✓	✓	✓	✓	✓					✓	✓				✓	✓	✓
SNPs3D		✗	✓	✓	✓					✓		✓						
SuSPect (disease-susceptibility-based SAV phenotype prediction)		✗	✓	✓	✓							✓					✓	
VEST3		✗	✓	✓	✓	✓		✓		✓		✓			✓		✓	
VEST4		✗	✓	✓	✓	✓		✓		✓		✓			✓		✓	

See Supplementary Table 1 for additional details on outputs, thresholds, variant types covered, and references.

### Clinical application of in silico predictions

While primarily developed to support genomic research, in silico predictions have also been widely used by clinical diagnostic laboratories to supplement clinical data for variant classification. However, as the substantial discordancy between tools and high rates of false positive predictions has become more evident, greater caution has been applied. Indeed, in the 2015 ACMG/AMP framework for variant interpretation, it is recommended that evidence from in silico tools should only be used when multiple tools are concordant and only to provide supporting-level evidence toward assessment of pathogenicity or benignity [2]. Using a quantitative Bayesian translation of the ACMG/AMP framework, supplementary evidence equates to a likelihood ratio of only twofold [22]. Tools most widely used clinically include PolyPhen-2, SIFT, and MutationTaster, due in large part to their inclusion within commercially developed interfaces [2, 4, 6, 23].

### Large-scale assays of cancer susceptibility gene function

Reliable assays of cellular function that correlate well with clinical pathogenicity have long been awaited by those working in genetic variation interpretation. The majority of early published experimental assays feature only a handful of variants, have been conducted in a post hoc and/or piecemeal fashion and often fail on reproducibility. Leveraging improved capability in gene editing technology, data from robust, systematic, high-throughput saturation genome editing experiments have recently become available for key cancer susceptibility genes, which have been shown to correlate strongly with well-curated orthogonally generated clinical classifications (Supplementary Tables 2, 3, 4).

These new-generation, clinically validated functional assays provide large “fresh” truth sets for unbiased evaluation of in silico tools. We thus sought to evaluate against functional assays of *BRCA1*, *BRCA2*, *MSH2*, *PTEN*, and *TP53* individually and in combination, predictive performance for 45 widely used in silico tools (72 tool–threshold combinations).

## MATERIALS AND METHODS

### Generation of functional truth sets of *BRCA1*, *BRCA2*, *MSH2*, *PTEN*, and *TP53* variants

For *BRCA1*, we used data on 2,321 nonsynonymous variants generated by Findlay et al. in which *BRCA1* function was assessed via assay of cellular fitness of HAP1 for the 13 exons comprising the RING and BRCT functional domains generated via saturation genome editing [24]. For *BRCA2* function, we used data generated by Couch et al., who performed a homology-directed DNA break repair (HDR) assay in *BRCA2*-deficient cells, assessing 237 variants in the *BRCA2* DNA-binding domain introduced via site-directed mutagenesis [25–28]. For *MSH2,* we used data for 5,212 single base substitution variants introduced by saturation mutagenesis from HAP1 survival following treatment with 6-TG, which induces lesions unrepairable by the MMR machinery [29]. For *PTEN* we integrated data for 7,244 variants generated on phosphatase activity in an artificial humanized yeast model with data from Variant Abundance by Massively Parallel Sequencing (VAMP-seq) in which *PTEN* protein expression in a human cell line was quantified for 4,112 *PTEN* variants, from which 2,380 nonsynonymous variants overlapped with the phosphatase activity data [30, 31]. As per specification of the ClinGen*TP53* expert group for clinical variant classification, we integrated data from (1) yeast-based transactivation assays performed eightfold for variants introduced by site-directed mutagenesis and (2) survival of isogenic *TP53*-wild-type and *TP53*-null cell populations treated with Nutlin-3 and/or etoposide for variants generated using Mutagenesis by Integrated TilEs (MITE), from which there were 2,314 overlapping variants [32–35].

Each gene-specific functional truth set was curated to include only missense variants, described in accordance to HGVS nomenclature for GRCh37 transcripts ENST00000357654 (*BRCA1*), ENST00000380152 (*BRCA2*), ENST00000233146 (*MSH2*), ENST00000371953 (*PTEN*), and ENST00000269305 (*TP53*). The potentially spliceogenic exonic variants at the two bases flanking the intron–exon boundary were also excluded. Missense variants were classified as nonfunctional (deleterious, DEL) or functional (tolerated, TOL) in accordance with functional assay specifications (Supplementary Table 2). Variants with results discordant between constituent assays (*PTEN* and *TP53*) were excluded from the functional truth sets, as were variants with intermediate assay activity for *BRCA1*, *BRCA2*, and *MSH2*. Of 12,624 nonsynonymous variants for which assay data were available, 11,212 were missense in suitable regions, of which 9,436 gave results of deleterious/tolerated (1,641 in *BRCA1*, 188 in *BRCA2*, 4,783 in *MSH2*, 957 in *PTEN*, and 1,867 in *TP53*). 1,413 variants were nonfunctional (deleterious) and 8,023 were functional (tolerated) (Supplementary Table 3).

### Generation of ClinVar truth-sets of *BRCA1*, *BRCA2*, *MSH2*, *PTEN*, and *TP53* variants

We also assembled available ClinVar classifications for these 9,436 missense variants, retaining those with ClinVar classifications of pathogenic/likely pathogenic (267 variants) or benign/likely benign (≥1 star rating) (66 variants). These were assigned in the ClinVar truth set as deleterious (DEL) and tolerated (TOL) respectively (Supplementary Table 4).

Tool evaluations were primarily focused on the functional truth sets, as many of the tools had been trained/evaluated using ClinVar data and/or tool predictions constituting part of the ClinVar classification.

### In silico tools

Forty-five in silico tools were selected on the basis of inclusion in publicly available/commercial variant interpretation resources and/or reported use in clinical diagnostics (Table 1, Supplementary Table 1) [2, 20, 21]. The parameters/thresholds for tool predictions as deleterious (DEL) or tolerated (TOL) were based on default author recommendations (Supplementary Table 5). Where there was variation from default author recommended settings reported in the literature or commonly used in practice, additional tool–threshold combinations were included, resulting in 72 in total. For example, for REVEL we specified three tool–threshold combinations: Revel_a: <0.4 predicted-TOL; > 0.7 predicted-DEL, Revel_b:  ≤0.7 predicted-TOL;  >0.7 Predicted-DEL, Revel_c: ≤0.5 predicted-TOL;  > 0.5 predicted-DEL. 57/72 tool–threshold combinations involved binary categorization above or below a cutoff; 15/72 were nonbinary, involving exclusion of an indeterminate scoring set of variants. We excluded from subsequent analysis tool–threshold combinations for which predictions (1) produced no discrimination (one exclusion: Integrated_fitCons_b: all calls deleterious), (2) were generated for <25% of variants examined (one exclusion: SNPs3D [calls for <3% of variants]). Following exclusions, we examined 70 tool–threshold combinations in total representing 44 tools. We also examined under a full concordancy model (i.e., discordant calls were excluded) for (1) pairwise combination 12 of the tools with best balanced accuracy and (2) three-way combination of (a) SIFT, PolyPhen-2 (HumVAR), and MutationTaster and (b) Revel b, PMut, and rfPred (Supplementary Table 5).

### Statistical analysis

Tool predictions were generated as per resources/versions specified in Supplementary Table 5. These predictions were compared to five gene-specific functional truth sets, the combined functional truth set of 9,436 variants (ALL) and the ClinVar truth set of 333 variants. For each of the 70 tool–threshold combinations, predictions of DEL, TOL, or missing were generated for each of the 9,436 missense variants. Missing predictions resulted in diminution of the total number of predictions where (1) the tool failed to make a prediction for the variant (indeterminate) and (2) the prediction lay in the range between the defined thresholds for TOL or DEL (indeterminate, e.g., score range 0.4–0.7 for Revel_a). Each prediction was assigned true positive (TP) where predicted-DEL and classified DEL in the truth set, true negative (TN) where predicted-TOL and classified TOL in the truth set, false positive (FP) where predicted-DEL and classified TOL in truth set, or false negative (FN) where predicted-TOL and classified DEL in truth set recall (Supplementary Table 6). For each functional truth set, the overall prevalence, detection prevalence, sensitivity (recall), specificity, positive predictive value (PPV, precision), and negative predictive value (NPV) were calculated. Balanced accuracy (BA), which combines sensitivity and specificity, was presented as the primary pan-performance metric [36]. We also calculated the Matthews correlation coefficient (MCC), which combines TP, TN, FP, and FN, the area under the curve (AUC), and the F1, which combines precision and recall (Supplementary Tables 7, 8) [37]. To adjust for differing contribution of the five gene-specific functional truth sets, the mean of the five outputs was also generated.

Positive likelihood ratios (PLRs) were generated comparing values above the threshold to those below; negative likelihood ratios (NLRs) were generated by comparing values below the threshold to those above (Supplementary Table 9). For REVEL and Meta-SNP, we undertook a banded analysis, examining the PLRs  for pathogenicity for various scoring bands above 0.7 against tool prediction <0.7; for PLRs for benignity (NLRs for pathogenicity), we examined various scoring bands below 0.7 and compared each to tool prediction >0.7 [38]. Where zero fields precluded generation of a PLR/NLR, we performed a Haldane correction (addition of 0.5 to each cell) (Supplementary Tables 10, 11).

To determine the optimal cutoff value for each tool, we used as the reference metric balanced accuracy (BA) calculated using the dichotomized scores, and iterated in 2% intervals from the lowest value. The optimization process was terminated when the tested cutoff value became higher than the maximum variant effect score of the tool evaluated. We then took the mean of the optimized thresholds for the five functional truth sets. We evaluated BA against this mean threshold for the six truth sets (five gene-specific and the combined functional truth set [ALL]).

Analyses were performed using R v.3.6.2 and STATAv15 (Timberlake Analytics).

## RESULTS

### Variant inclusion

We used 9,436 variants in the combined functional truth set which overall had a sensitivity of 0.91 and specificity of 0.95 for ClinVar calls (Supplementary Table 4). We included 70 tool–thresholds representing 44 tools. Of these, 9/70 tool–threshold combinations generated predictions for <80% of the variants (variant inclusion, Supplementary Table 8). For example, for Revel_a, the threshold setting recommended for clinical application in the UK Association for Clinical Genomic Science (UK-ACGS) guidance, scores of <0.4 are predicted as tolerated and scores of >0.7 are predicted as deleterious. For Revel_a, 42.4% of the 9,436 variants score 0.4–0.7 such that they are indeterminate and not classified [39, 40]. Notably, within this indeterminate REVEL range, while the true positives cluster toward the higher end, the distribution for the true negatives is relatively even (Supplementary Fig. 1).

### Overall performance

The true prevalence of deleterious variants in the combined functional truth set was 15% (1,413/9,436). However, the detection prevalence (i.e., the total proportion *called* by the in silico tool as deleterious) was >50% for 56/70, >75% for 28/70, and >90% for 11/70 of the tool–threshold combinations. Thus, while sensitivity was generally high (>80% for 56/70 tool–threshold combinations) this tended to be at a cost of poor specificity and PPV.

**Fig. 1:**
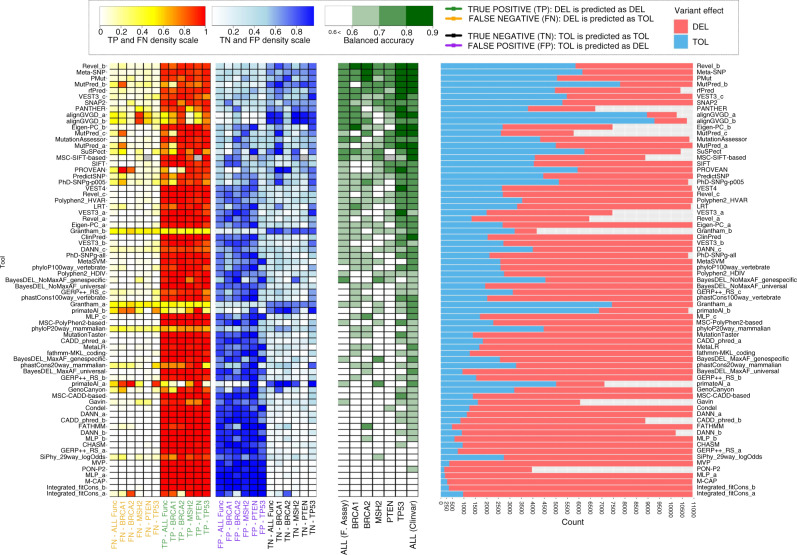
Balanced accuracy for 70 tool–threshold combinations for seven truth sets. Rates of true positive (TP), false negative (FN), true negative (TN), and false positive (FP) tool calls against functional truth sets also shown, along with rates of tool calls of deleterious (DEL, pink), tolerated (TOL, blue) or indeterminate (gray).

Based on mean BA across the five gene-specific truth sets, the best performing tool–threshold combinations were metatools Revel_b and Meta-SNP. Revel_b (tolerated ≤ 0.7, deleterious >0.7) exhibited BA = 79%, reflecting sensitivity of 89% and specificity of 68% and Meta-SNP (tolerated ≤ 0.5, deleterious >0.5) exhibited BA = 79%, reflecting sensitivity of 92% and specificity of 66% (Fig. 1 and Supplementary Table 5). Also strongly performing were PMut, MutPred_b, and metatools rfPred and VEST3_c (tolerated ≤ 0.5; deleterious >0.5). Strong performances for some tool–threshold combinations, such as PANTHER and Eigen-PC_b (tolerated < 0; deleterious >0.5) must be caveated by their levels of variant exclusion (54 and 31% respectively). The tools most widely used clinically, SIFT, PolyPhen2 (HumVar), and MutationTaster ranked in positions 17th, 23rd, and 45th for BA: their high sensitivities (96–98%) came at the cost of poorer specificities (20–38%) (Supplementary Table 8).

Tool performance for variants excluded due to being in the intermediate range of the assays for *BRCA1*, *BRCA2*, and *MSH2* is shown in Supplementary Fig. 2 and Supplementary Table 12. Median scores for the functionally intermediate variants largely lay between medians for the deleterious and tolerated group, but with little evidence of graded correlation.

### Consistency between gene-specific truth sets

The five individual-gene functional truth sets varied in data set size, gene-representativeness, and proportion of deleterious variants. The 4,783 *MSH2*, 957 *PTEN*, and 1,867 TP53 variants included spanned the full gene, while the 1,641 *BRCA1* were restricted to the RING/BRCT domains and the 188 *BRCA2* variants likewise all lay within the DNA-binding domain. Interestingly, examination for all missense variants across the *BRCA1* gene showed a higher median REVEL score for variants outside of any domain (0.57) than for variants within the BRCT domain (0.48) (Supplementary Fig. 3). The proportion of deleterious variants in the *MSH2* data set (8%) was much lower than for *BRCA1* (23%), *PTEN* (20%), and *TP53* (22%) and in particular *BRCA2* (34%); this metric influences PPV and NPV. Based on ordinal rankings for mean BA, there was broad consistency across the five individual-gene functional truth sets for tool–threshold combinations with binary cutoffs (Supplementary Table 8). There was greater heterogeneity across the five individual-gene functional data sets for tools with nonbinary thresholds, on account of the proportion of TP/TN excluded in the indeterminate range.

### Predictions for loss-of-function versus dominant-negative effects

Pathogenic missense variants in *BRCA1*, *BRCA2*, and *MSH2* are understood largely to act via a two-hit (loss-of-function) mechanism. For variants in *PTEN* and *TP53*, pathogenic effect can be conferred by either loss-of-function or dominant-negative (gain-of-function) effect. For *TP53*, performance on the Nutlin-tp53WT assay would be predicted to select for variants acting by dominant-negative effect (DNE).

The BA for *TP53* was above the mean BA for the five gene-specific truth sets for all 20/20 of the top performing tools. For *BRCA1*, *BRCA2*, *MSH2*, and *PTEN* this proportion was 11/20, 8/20, 6/20, and 9/20 respectively. Additionally, for each of *TP53* and *PTEN*, REVEL scores for variants at the known dominant-negative hotspots exceeded the median scores across all other deleterious variants (Supplementary Fig. 4). Moreover, across the *TP53* variant set, there was strong correlation (*p* < 2.2 × 10^–16^) between the REVEL score and the p53WT Nutlin-3 *z*-score (Supplementary Fig. 5).

### Combinations of tools

Despite Revel_b being a metapredictor encompassing twelve component tools, its mean BA across the five genes could be improved from 79% to up to 84% by concordance combination with other high-performing tools such as Meta-SNP, VEST3, rfPred, and MutPred, although this led to dropout of discordant variants ranging from 6% to 26% (Supplementary Table 8).

### Performance against ClinVar

The prediction parameters for the 70 tool–threshold combinations against the ClinVar truth set generally exceed performance against the mean of the functional truth sets, likely reflecting the direct or indirect relationship between ClinVar classifications and tool training [11]. Overall, there was consistency in the ordinal performance of most tools between the mean of the functional truth sets and the ClinVar truth set, with Revel_b ranking second for BA against the ClinVar truth set. Performance against the ClinVar truth set appeared disproportionately better for tools trained exclusively on ClinVar, such as ClinPred, compared to tools trained on different, mixed data sets.

### Positive and negative likelihood ratios

Particularly relevant metrics for clinical classification are the positive likelihood ratio for calling deleterious (true positive rate/false positive rate) and NLR for calling deleterious (or PLR for calling benignity, the true negative rate/false negative rate). Tool–threshold combinations performing well on BA tended to exhibit strong but balanced PLRs/NLRs, for example Revel_b had mean PLR = 3.13 (2.75–3.58) and NLR = 7.20 (6.27–8.33) while Meta-SNP exhibited PLR = 2.79 (2.49–3.14) and NLR = 9.98 (8.81–11.3) (Supplementary Table 9).

Tool–threshold combinations with high sensitivity and low specificity typically exhibited poor PLR but strong NLR, driven by low rates of false negatives. Tool–threshold combinations with high specificity but weaker sensitivity exhibited poor NLR but much stronger PLR, driven by lower rates of false positives. Using the mean of the five functional truth sets, PLRs and NLRs at different thresholds of REVEL and Meta-SNP were calculated (Table 2, Supplementary Tables 10, 11).

## DISCUSSION

We present predictive parameters and positive/negative likelihood ratios for 44 in silico tools and 70 tool–threshold combinations, examining 9,436 missense variants generated from systematic functional assays for five genes, which have been validated against clinical pathogenicity.

We demonstrated that most widely used in silico tools have high sensitivity, that is they are unlikely to miscall a truly deleterious variants. However, many of the tools maintain high sensitivity at the expense of very high false positive call rates, as reflected by the 56/70 tools which called more than 50% of the variants as deleterious (true frequency 15%). For the tools widely used in clinic at their specified thresholds, across the five functional truth sets mean PPV was 30% for SIFT, 28% for Polyphen2 (HumVar) and 26% for MutationTaster [11].

Because tools are generally calibrated to overcall as pathogenic, their negative predictive value is typically good: 44/70 tool–threshold combinations had NPV > 95%. Furthermore, NPV is dependent on the prevalence of true pathogenic variants; the NPV would further improve in the context of a clinical laboratory in which prevalence of true pathogenic variants is typically lower than the 15% in the combined functional truth set. These data argue against current equivalence within the ACMG/AMP framework for in silico tool prediction of pathogenicity and benignity. These data replicate similar observations reported in previous analyses using ClinVar truth sets [17]. For example, 2,361 variants have REVEL score <0.5: of these, 2,328 are true negatives and only 33 are false negatives.

As tool thresholds are typically set for high sensitivity, it is the corresponding specificity which drives our rankings for BA. At specified thresholds, Revel_b, Meta-SNP, PMut, MutPred_b, rfPred and VEST3_c all perform well, with BA ≥ 73%, AUC ≥ 83%, and MCC ≥ 41%. Notably REVEL, Meta-SNP and rfPred are all metapredictors, that is they have been developed using machine learning optimized amalgamation of component algorithms (Supplementary Table 1).

Although included as it is a widely used tool, we would caveat generalizability of performance of Align-GVGD, as not only have sequence alignments been especially well curated for the genes analyzed, but the tool was trained on *BRCA1/2* classifications and *TP53* functional data sets [32, 41].

Against the five functional truth sets, for concordant calls of deleterious for SIFT, PolyPhen2_HumVar, and MutationTaster, the mean positive likelihood ratio is only 1.21 (1.16–1.27), with 39% of variants dropping out due to discordant calls (Supplementary Table 9) [11]. More broadly, for any nonbinary tool–threshold combinations or combining of tools using a concordance model, any apparent boost in calculated BA must be caveated by the inevitable exclusion of a substantial proportion of the “difficult' indeterminate/discordant variants. As the 2015 ACMG/AMP framework does not specify which in silico tools are allowable, it is duly conservative in offering only supplementary evidence weighting (likelihood ratio ~2) and only where multiple tools are concordant. Using REVEL at the dichotomous threshold of 0.7 offers PLR of 3.13 (2.75–3.58) and NLR of 7.20 (6.27–8.33), but higher evidence weighting may be warranted for scores at the extreme tails. For example the LR of 6.74 (5.24–8.82) for REVEL or 42.9 (14.4–406) for Meta-SNP for scores of 0.8–1.0 would comfortably constitute stronger evidence toward pathogenicity, as would the LR of 34.3 (31.5–37.3) for REVEL or 19.4 (15.6–24.9) for Meta-SNP for scores of <0–0.4 toward benignity (Table 2) [22, 42]. Our data would thus overall support calibrated use of high-performing metatools for clinical variant interpretation, rather than ad hoc combinations of multiple tools. Provided the tool has not been trained on the functional data, as in silico predictions are derived from orthogonal data to functional assays, we would support the two evidence types being separately counted toward a variant classification.

**Table 2:** Positive likelihood ratios (PLR) and negative likelihood ratios (NLR) for pathogenicity for REVEL and Meta-SNP predictions using binary thresholds and score bands, examining the mean of results for each gene for *BRCA1*, *BRCA2*, *MSH2*, *PTEN*, and *TP53* and the unweighted result for all variants (see also Supplementary Tables 10 and 11).

		REVEL				Meta-SNP			
		Mean		All		Mean		All	
		PLR	NLR	PLR	NLR	PLR	NLR	PLR	NLR
Binary	0.9	45.9 (9.63–302.)	1.98 (1.25–5.67)	5.01 (4.59–5.48)	1.69 (1.54–1.84)	32.9 (3.08–470)	1.01 (0.085–26.2)	49.2 (14.9–162)	1.01 (0.308–3.36)
	0.8	6.24 (4.83–8.22)	4.05 (3.37–4.98)	3.15 (3.00–3.31)	3.02 (2.87–3.17)	23.5 (8.69–203)	1.40 (0.773–5.99)	15.2 (12.7–18.2)	1.38 (1.15–1.65)
	0.7	3.13 (2.75–3.58)	7.20 (6.27–8.33)	2.33 (2.26–2.41)	5.39 (5.21–5.57)	6.80 (5.27–9.09)	3.64 (2.87–4.77)	5.68 (5.33–6.06)	3.43 (3.22–3.66)
	0.5	1.49 (1.40–1.59)	19.9 (18.7–21.2)	1.37 (1.35–1.39)	12.4 (12.2–12.6)	2.79 (2.49–3.14)	9.98 (8.81–11.3)	2.75 (2.66–2.85)	8.62 (8.32–8.92)
	0.4	1.18 (1.14–1.23)	19.0 (18.2–19.7)	1.15 (1.13–1.16)	10.6 (10.4–10.7)	2.02 (1.86–2.20)	15.4 (14.1–16.8)	1.88 (1.84–1.93)	14.3 (14.0–14.7)
	0.1	1.00 (1.00–1.00)	1.87 (1.87–1.88)	1.00 (1.00–1.00)	16.7 (16.7–16.7)	1.05 (1.03–1.07)	25.1 (24.6–25.6)	1.02 (1.02–1.03)	41.7 (41.5–41.9)
Band	0.9–1.0	102 (17.9–701)	Compared to REVEL < 0.7	6.19 (5.74–6.67)	Compared to REVEL < 0.7	122. (10.8–1,799)	Compared to Meta-SNP < 0.7	157 (47.9–518)	Compared to Meta-SNP < 0.7
	0.8–1.0	6.74 (5.24–8.82)		3.14 (3.00–3.28)		42.9 (14.4–406.)		24.9 (21.0–29.5)	
	0.8–0.9	6.77 (5.07–9.15)		3.77 (3.50–4.06)		42.7 (14.2–406.)		24.6 (20.7–29.2)	
	0.7–0.8	3.07 (2.23–4.28)		2.93 (2.62–3.26)		6.64 (5.01–9.07)		5.62 (5.20–6.07)	
	0.5–0.7	Compared to REVEL > 0.7	7.44 (6.64–8.38)	Compared to REVEL > 0.7	4.95 (4.81–5.10)	Compared to Meta-SNP > 0.7	3.55 (2.87–4.51)	Compared to Meta-SNP > 0.7	3.19 (3.02–3.37)
	0.4–0.5		55.1 (50.0–60.7)		25.3 (24.8–25.8)		14.4 (11.1–19.1)		10.4 (10.0–11.0)
	0–0.5		30.4 (27.1–34.1)		16.8 (16.4–17.3)		10.6 (8.32–14.2)		8.92 (8.42–9.45)
	0–0.4		34.3 (31.5–37.3)		18.2 (17.9–18.6)		19.4 (15.6–24.9)		18.1 (17.1–19.1)

The relationship between functional assay results, clinical classifications, and true underlying pathogenicity remains elusive. Imperfect correlation of the assay data to clinical classification may in part reflect erroneous clinical classifications resident on public databases. Clinical classifications are indeed not sacrosanct and are only as good as the comprehensiveness and accuracy of available clinical information, as well as the validity of classification schema employed [43, 44]. The functional assays assessed are relatively recent; their incorporation into clinical classification, ClinVar, and other resources will further confound data benchmarking.

Inflation of tool performance against publicly available data sets (in particular ClinVar), overfitting and the shortcomings of clinically derived classifications have been well described previously [11]. Indeed, many truth sets previously used for tool evaluations (1) have overlapped with the data sets upon which tools were trained or (2) correlated poorly with true clinical pathogenicity, comprising population data and/or prokaryotic cell models and/or clinical classifications of poor quality [11, 45]. Thus, while the functional assays we have used are unlikely to perfectly recapitulate true human pathogenesis, given their powerful correlation against clinical classifications, size of data and systematic generation, arguably they represent leading truth sets for unbiased evaluation of tool performance.

Although we excluded the two intron-flanking exonic variants on account of potential spliceogenic effect, other spliceogenic exonic variants resulting in a null protein will have been called as deleterious by most assays: we have assumed this group to be small in number and roughly consistent between genes. Although our analysis has focused predominantly on missense variants for which pathogenesis is via loss of function, our data for *TP53/PTEN* support the tools discriminating comparably for DNE. This observation is consistent with previous reports in which prediction for DNE compared to loss-of-function variants was poorer for older tools (SIFT, Polyphen) but equivalent using newer algorithms such as REVEL [17, 18].

The observed variation in tool performance between the five individual-gene truth sets likely reflects heterogeneity in composition of pathogenic variants types (loss of function versus DNE), varying accuracy of assay in recapitulation of true pathogenicity, and sampling variation (chance). It has been argued that there will be systematic differences gene by gene in how tools perform, on account of innate gene-specific differences in the genomic context of pathogenic and benign variants. Data from a broader range of multiplex assays of variant effect (MAVEs) examining the full spectrum of coding variants would enable further exploration of such hypotheses. However, while there are rapid advances in technology for high-throughput gene editing and assay readout, expansion of MAVEs to additional genes has been limited on account of lack of availability of clinical truth sets by which to validate assays and limited understanding of mutational mechanism of clinical pathogenicity [46].

A perennial issue in the arena of variant interpretation is that of intermediate penetrance/effect. The clinical model of dichotomous classification as pathogenic or benign imperfectly accommodates underlying continuity of clinical penetrance and a corresponding more continuous distribution of in vivo and in vitro cellular function. To simplify the tool assessment, we removed from our functional truth sets all variants scored as intermediate for assay performance. However, better quantitation of variants of intermediate effect will require study of these intermediate assay scores and will necessitate use of continuous measurement rather than binary categories for both in silico predictions and functional assays.

Performance of combinations for high-performing tools indicates room for improvement in algorithms. Furthermore, while we focused predominantly on established/author-provided tool thresholds, generation of new thresholds optimized for BA against these functional data sets indicated potential for substantially improved tool performance, in particular for Meta-SNP and MutPred (Supplementary Table 13). Tools could be further evolved using more advanced machine learning approaches with weighting of contribution of these functional truth sets to optimize tool combination, performance, and variant inclusion.

There is a growing preponderance of in silico tools. As many previous authors have found, many of these tools used at their recommended thresholds have very poor specificity and PPV. Where ClinVar was also used to train the tools, tool evaluation against ClinVar may misrepresent performance due to overfitting. The cautious ACMG/AMP evidence weights may still be overly generous for many in silico tools. However, evaluation against a large systematically generated clinical grade truth set of functional assay data allows unbiased identification of the more predictive tools and discriminatory thresholds. Our data suggest that greater weights of evidence toward pathogenicity/benignity might be afforded for specific tools such as REVEL and Meta-SNP, with potential for evidence calibration by absolute score. Using a Bayesian conversion, the respective relevant positive and negative likelihood ratios can be incorporated into the ACMG/AMP framework [22, 42].

## Supplementary information

Supplementary information

Supplementary note

Supplementary tables

## Data Availability

The data analyzed are all publicly available per references/URLs provided. While this paper does not contain primary research data, materials and data developed during this study will be made available upon request to the corresponding author.
