# Supplementary material for: Clinical likelihood ratios and balanced accuracy for 44 in silico tools against multiple large-scale functional assays of cancer susceptibility genes
Source: Genet Med. 2021 Jul 6;23(11):2096–104. doi: 10.1038/s41436-021-01265-z (PMC8553612; doi:10.1038/s41436-021-01265-z)
Supplement: Supplementary file 1 — Supplementary information [file 41436_2021_1265_MOESM1_ESM.pdf]

Supplementary Figures

Clinical likelihood ratios and balanced accuracy for 44 in silico tools AGAINST multiple large-scale functional assays of cancer susceptibility genes

C. Cubuk[1]*, A. Garrett[1]*, S. Choi*[1], L. King[1], C. Loveday[1], B. Torr[1], G.J. Burghel[2,] M. Durkie[3], A. Callaway[4, 5], R. Robinson[6], J. Drummond[7], I. Berry[6], A. Wallace[2], D. Eccles [5,8], M. Tischkowitz ,[7,19], N. Whiffin [10,11,12], J.S. Ware [11,12,] H. Hanson[1, 13], C. Turnbull [1, 14] and CanVIG-UK

*these authors contributed equally to the work

[1] Division of Genetics and Epidemiology, Institute of Cancer Research, Sutton, UK

[2] Manchester Centre for Genomic Medicine and NW Laboratory Genetics Hub, Manchester University NHS Foundation Trust, Manchester, UK

[3] Sheffield Diagnostic Genetics Service, Sheffield Children's NHS Foundation Trust, Sheffield, UK

[4] Wessex Regional Genetics Laboratory, Salisbury Hospital NHS Foundation Trust, Salisbury, UK

[5] Human Genetics and Genomic Medicine, Faculty of Medicine, University of Southampton, Southampton, UK

[6] Yorkshire and North East Genomic Laboratory Hub, Leeds Teaching Hospitals NHS Trust, Leeds, UK

[7] East Genomic Laboratory Hub, Cambridge University Hospitals Genomic Laboratory, Cambridge University Hospitals, Cambridge, UK

[8] Cancer Sciences, Faculty of Medicine, University of Southampton, Southampton, UK

[9] Department of Medical Genetics, National Institute for Health, Research Cambridge Biomedical Research Centre, University of Cambridge, Cambridge, UK

[10] Cardiovascular Genetics and Genomics, National Heart and Lung Institute, Imperial College London, London, UK

[11] NIHR Royal Brompton Cardiovascular Biomedical Research Unit, Royal Brompton & Harefield Hospitals & Imperial College London, London, UK;

[12] The Wellcome Centre for Human Genetics, Oxford, UK

[13] Department of Clinical Genetics, St. George's University Hospitals NHS Foundation Trust, London, UK

[14] Cancer Genetics Unit, Royal Marsden NHS Foundation Trust, London, UK

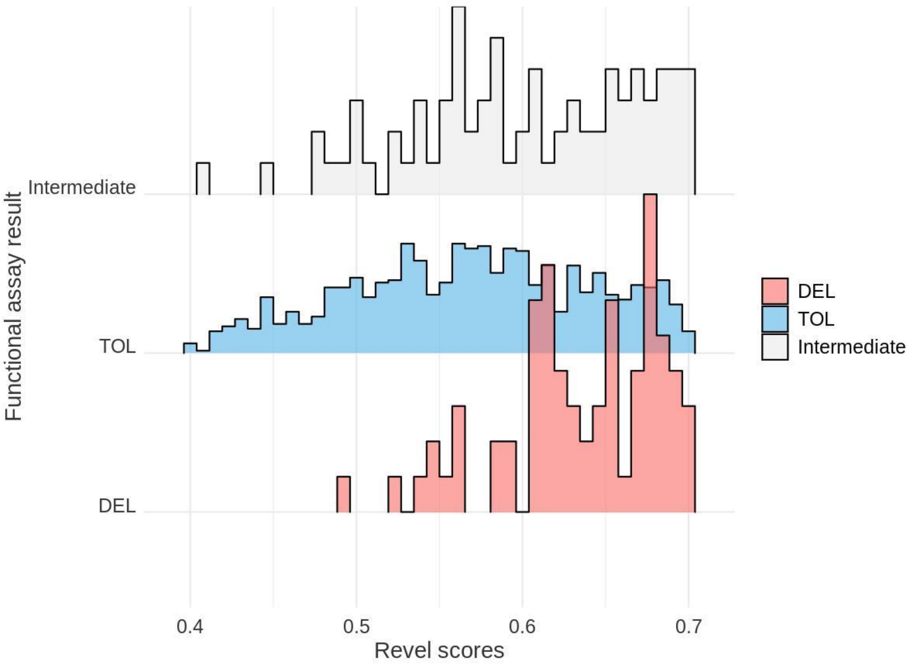

Supplementary Figure 1: Distribution of Revel Scores 0.4-0.7 for BRCA1 variants by functional assay group, deleterious (70 variants), tolerated (1016 variants) or intermediate (79 variants)

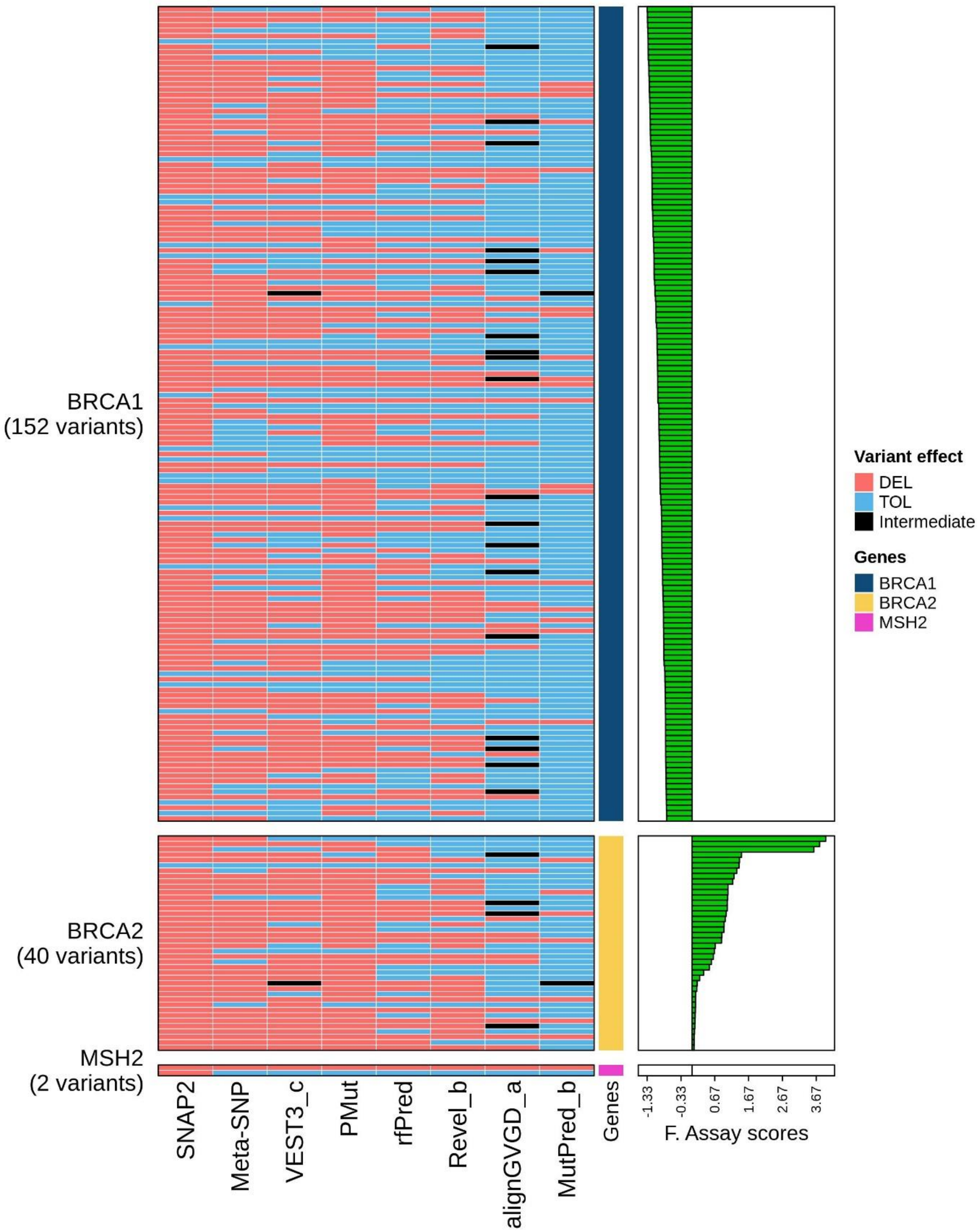

Supplementary Figure 2: Examination of in silico predictions variants scoring in the intermediate range of functional assays for BRCA1, BRCA2 and MSH2 for 8 high-performing tools. Variants are ordered top-to-bottom for their score on the functional assay, with those at the top end being variants scoring nearest to the threshold for being called as deleterious

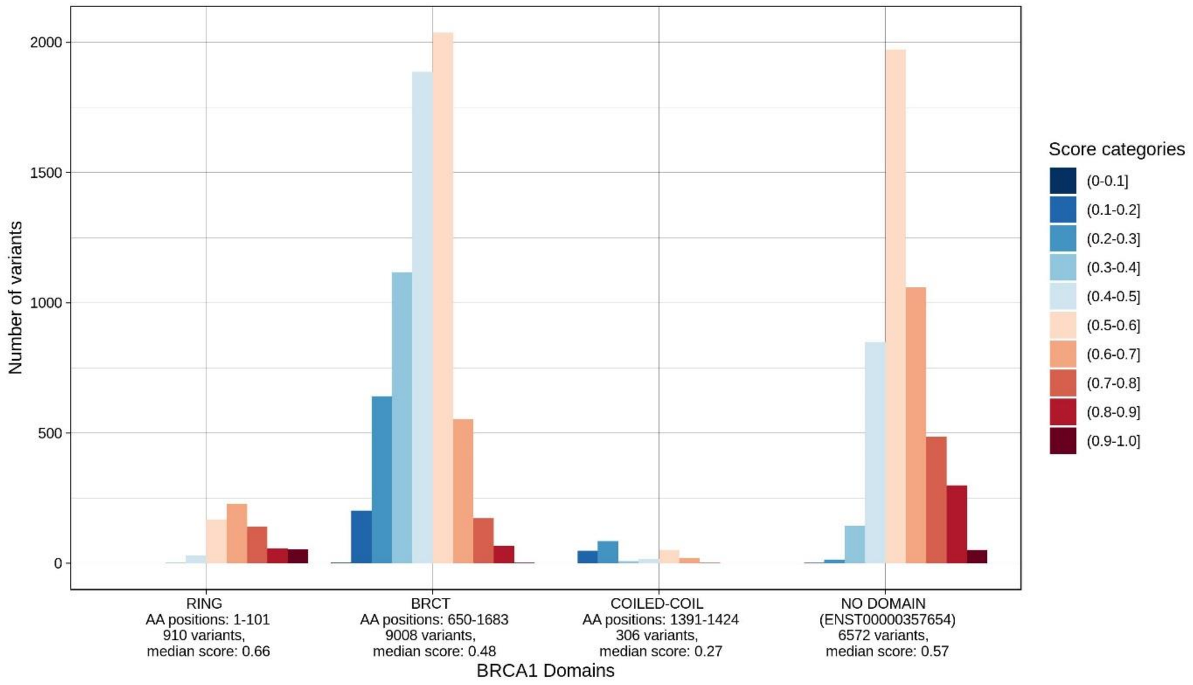

Supplementary Figure 3: Distribution by gene domain of Revel Scores for all missense BRCA1 variants (including variants with and without assay data)

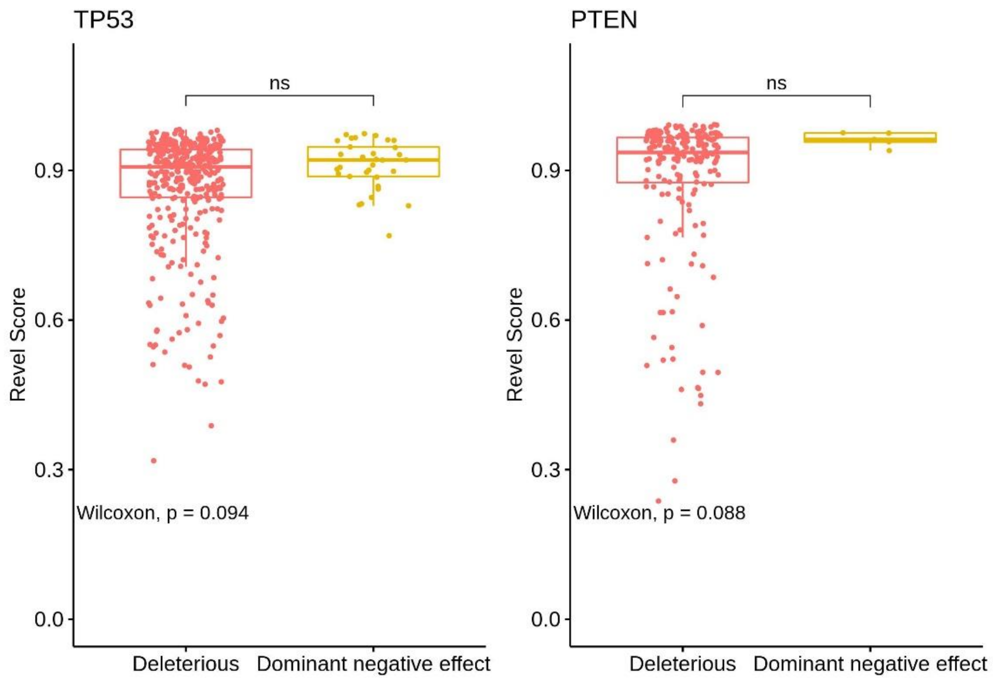

Supplementary Figure 4: Comparison of Revel scores against all other variants classified by assay as deleterious for variants of established dominant negative effect in TP53 (missense at codons 175, 245, 248, 249, 273 and 282) and PTEN (p.Pro38Ser, p.Cys124Ser, p.Arg130Gly, p.Arg130Gln, p.Gly129Glu)

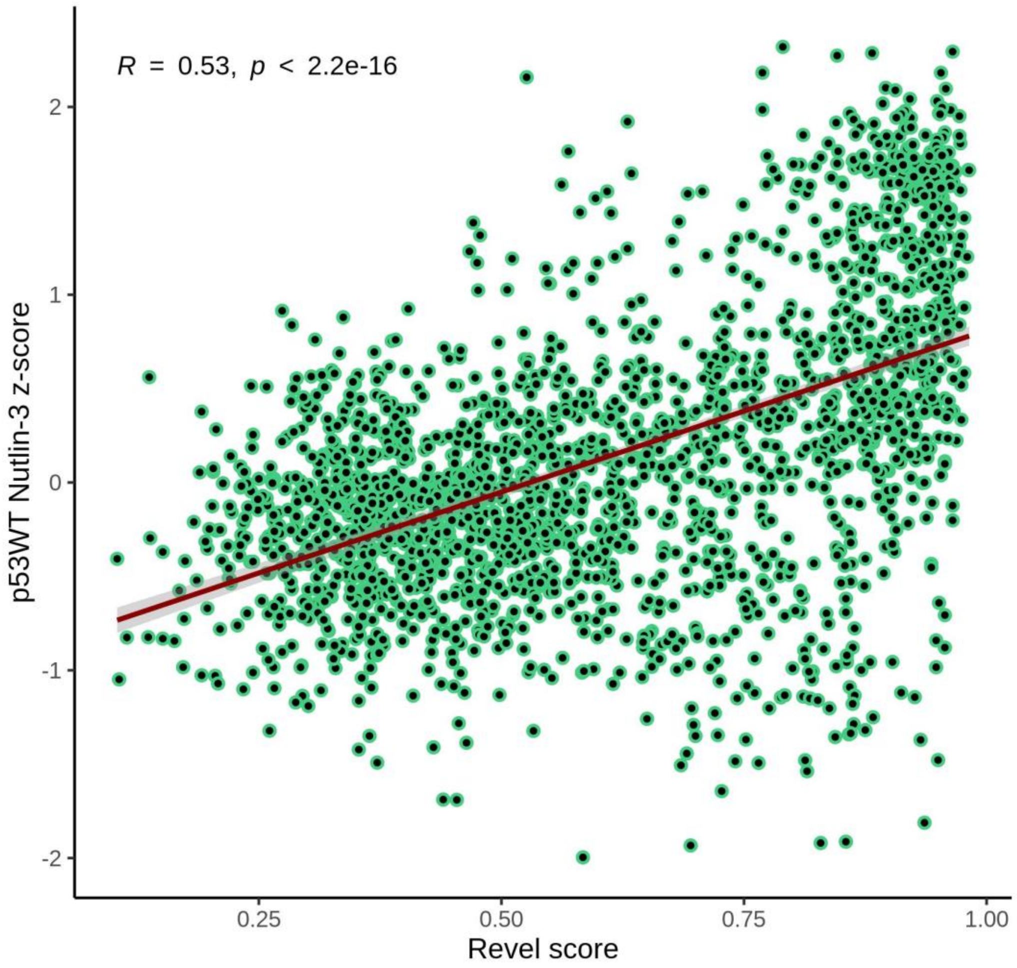

Supplementary Figure 5: Correlation of p53WT Nutlin-3 z-score (an assay of dominant negative action) against Revel Score for the 1867 TP53 variants included in the analysis
