# Supplementary material for: Clinical likelihood ratios and balanced accuracy for 44 in silico tools against multiple large-scale functional assays of cancer susceptibility genes
Source: Genet Med. 2021 Jul 6;23(11):2096–104. doi: 10.1038/s41436-021-01265-z (PMC8553612; doi:10.1038/s41436-021-01265-z)
Supplement: Supplementary file 2 — Supplementary note [file 41436_2021_1265_MOESM2_ESM.docx]

# Supplementary NOte

# CanVIG-UK

AUTHORS

C. Cubuk^1^, A. Garrett^1^, S. Choi^1^, L. King^1^, G.J. Burghel^2 ,^  M. Durkie^3^, A. Callaway^4^, R. Robinson^5^, J. Drummond^6^, A. Wallace^2^, M. Alikian^1^, D. Eccles^7^, M. Tischkowitz^8^, H. Hanson^9^, and C. Turnbull^1^.

^1^ Division of Genetics and Epidemiology, Institute of Cancer Research, Sutton, London, UK.

^2^ Manchester Centre for Genomic Medicine and NW Laboratory Genetics Hub, Manchester University NHS Foundation Trust, Manchester, UK.

^3^ Sheffield Diagnostic Genetics Service, Sheffield Children's NHS Foundation Trust, Sheffield, UK.

^4^ Wessex Regional Genetics Laboratory, Salisbury Hospital NHS Foundation Trust, Salisbury, UK.

^5^ Yorkshire Regional Genetics Service, Leeds Teaching Hospitals NHS Trust, Leeds, UK.

^6^ East Anglian Medical Genetics Service, Cambridge University Hospitals NHS Foundation Trust, Cambridge, UK.

^7^ Cancer Sciences Research Group, University of Southampton Faculty of Medicine, Southampton, UK.

^8^ Department of Medical Genetics and National Institute for Health Research Cambridge Biomedical Research Centre, University of Cambridge, Cambridge, Cambridgeshire, UK.

^9^ Department of Clinical Genetics, St. George's University Hospitals NHS Foundation Trust, London, UK.

# CanVIG-UK

NON-AUTHOR CONTRIBUTORS

S. Samant^32^ , A. Lucassen^58^ , A. Znaczko^43^, A. Shaw^22^ , A. Ansari^33^ , A. Kumar^20^, A. Donaldson^53^ , A. Murray^18^ , A. Ross^17^ , A. Taylor-Beadling^21^ , A. Taylor^17^ , A. Innes^24^ , A. Brady^28^ , A. Kulkarni^22^ , A.-C. Hogg^3^ , A. Ramsay Bowden^17^ , A. Hadonou^48^ , B. Coad^47^ , B. McIldowie^18^ , B. Speight^17^ , B. DeSouza^48^ , B. Mullaney^5^ , C. McKenna^12^ , C. Brewer^43^ , C. Olimpio^17^ , C. Clabby^39^ , C. Crosby^48^ , C. Jenkins^41^ , C. Armstrong^32^ , C. Bowles^44^ , C. Brooks^21^ , C. Byrne^12^ , C. Maurer^6^ , D. Baralle^58^ , D. Chubb^1^ , D. Stobo^33^ , D. Moore^34^ , D. O'Sullivan^32^ , D. Donnelly^12^ , D. Randhawa^23^ , D. Halliday^40^ , E. Atkinson^51^ , E. Baple^19^ , E. Rauter^23^ , E. Johnston^37^ , E. Woodward^2,29^ , E. Maher^8^, E. Sofianopoulou^16^ , E. Petrides^41^ , F. Lalloo^2^ , F. McRonald^42^ , F. Pelz^52^ , I. Frayling^18^ , G. Evans^2,29^ , G. Corbett^12^ , G. Rea^12^ , H. Clouston^3^ , H. Powell^30^ , H. Williamson^53^ , H. Carley^48^ , H.J.W. Thomas^25^, I. Tomlinson^13^ , J. Cook^46^ , J. Hoyle^20^ , J. Tellez^31^ , J. Whitworth^17^ , J. Williams^50^ , J. Murray^34^ , J. Campbell^26^ , J. Tolmie^32^ , J. Field^37^ , J. Mason^14^ , J. Burn^30^ , J. Bruty^17^ , J. Callaway^4^ , J. Grant^33^ , J. Del Rey Jimenez^48^ , J. Pagan^34^ , J. VanCampen^23^ , J. Barwell^54^ , K. Monahan^28^ , K. Tatton-Brown^47^ , K.-R. Ong^13^ , K. Murphy^32^ , K. Andrews^17^ , K. Mokretar^22^ , K. Cadoo^49^ , K. Smith^53^ , K. Baker^4^ , K. Brown^23^ , K. Reay^14^ ,K. McKay Bounford^33^ , K. Bradshaw^37^ , K. Russell^15^ , K. Stone^22^ , K. Snape^47^ , L. Crookes^3^ , L. Reed^20^ , L. Taggart^12^ , L. Yarram^15^ , L. Cobbold^48^ , L. Walker^38^ , L. Walker^40^ , L. Hawkes^47^ , L. Busby^21^ , L. Izatt^22^ , L. Kiely^21^ , L. Hughes^14^ , L. Side^57^ , L. Sarkies^17^ , K.-L. Greenhalgh^27^, M. Shanmugasundaram^13^ , M. Duff^39^ , M. Bartlett^28^ , M. Watson^3^ , M. Owens^44^ , M. Bradford^55^ , M. Huxley^14^ , M. Slean^32^ , M. Ryten^22^ , M. Smith^56^ , M. Ahmed^20^ , N. Roberts^2^ , C. O'Brien^51^ , O. Middleton^32^ , P. Tarpey^6^ , P. Logan^12^ , P. Dean^5^ , P. May^23^ , P. Brace^20^ , R. Tredwell^37^ , R. Harrison^36^ , R. Hart^13^ , R. Kirk^3^ , R. Martin^30^ , R. Nyanhete^3^ , R. Wright^2^, R. Martin^12^ , R. Davidson^33^ , R. Cleaver^43^ , S. Talukdar^47^ , S. Butler^14^ , J. Sampson^18^ , S. Ribeiro^50^ , S. Dell^46^ , S. Mackenzie^31^ , S. Hegarty^12^ , S. Albaba^3^ , S. McKee^35^ , S. Palmer-Smith^18^ , S. Heggarty^12^ ,S. Ellard^44^ , S. MacParland^12^ , S. Greville-Heygate^59^ , S. Daniels^4^ ,S. Prapa^17^ ,S. Abbs^6^ , S. Tennant^32^ , S. Hardy^42^ , S. MacMahon^50^ , T. McVeigh^50^ , T. Foo^50^ , T. Bedenham^41^ , T. Cranston^41^ , T. McDevitt^39^ , V. Clowes^28^ , V. Tripathi^22^ , V. McConnell^12^ , N. Woodwaer^45^ , Y. Wallis^14^ , Z. Kemp^50^, G. Mullan^12^ , L. Pierson^12^, L. Rainey^12^ and C. Joyce^60^.

^12^ Belfast Health & Social Care Trust, Belfast, UK

^13^ Birmingham Women’s and Children’s NHS Foundation Trust, Birmingham, UK

^14^ Central and South Genomic Laboratory Hub, Birmingham Women’s and Children’s NHS Foundation Trust, Birmingham, UK

^15^ Bristol Genetics Laboratory, North Bristol NHS Trust, Bristol, UK

^16^ Public Health and Primary Care, Clinical Medicine, University of Cambridge, Cambridge, UK

^17^ Cambridge University Hospitals NHS Foundation Trust, Cambridge, UK

^18^ Institute of Medical Genetics, University Hospital of Wales, Cardiff and Vale University Health Board, Cardiff, UK

^19^ Genomics England, London, UK

^20^ Great Ormond Street Hospital for Children NHS Foundation Trust, London, UK

^21^ North Thames Genomic Laboratory Hub, Great Ormond Street Hospital for Children NHS Foundation Trust, London, UK

^22^ Department of Clinical Genetics, Guy’s and St Thomas’ NHS Foundation Trust, London, UK

^23^ South East Genomic Laboratory Hub, Guy’s and St Thomas’ NHS Foundation Trust, London, UK

^24^ Genomic Medicine Service, Imperial College Healthcare NHS Trust, London, UK

^25^ Faculty of Medicine, Department of Surgery & Cancer, Imperial College London, London, UK

^26^ Institute of Neurology, UCL Queen Square Institute of Neurology, London, UK

^27^ Liverpool Women’s NHS Foundation Trust, Liverpool, UK

^28^ London North West University Healthcare NHS Trust, London, UK

^29^ Division of Evolution and Genomic Sciences, School of Biological Sciences, Faculty of Biology Medicine and Health, The University of Manchester, Manchester, UK.

^30^ The Newcastle upon Tyne Hospitals NHS Foundation Trust, Newcastle upon Tyne, UK

^31^ North East and Yorkshire Genomic Laboratory Hub, The Newcastle upon Tyne Hospitals NHS Foundation Trust, Newcastle upon Tyne, UK

^32^ NHS Grampian, Aberdeen, UK

^33^ NHS Greater Glasgow and Clyde, Glasgow, UK

^34^ NHS Lothian, Edinburgh, UK

^35^ Northern Ireland Regional Genetics Service, Belfast Health & Social Care Trust, Belfast, UK

^36^ Nottingham University Hospitals NHS Trust, Nottingham, UK

^37^ East Midlands and East of England Genomics Laboratory, Nottingham University Hospitals NHS Trust, Nottingham, UK

^38^ University of Otago, Otago, New Zealand

^39^ Our Lady's Children's Hospital, Crumlin, Dublin, Ireland

^40^ Clinical Genetics, Oxford University Hospitals NHS Foundation Trust, Oxford, UK

^41^ West Midlands, Oxford and Wessex Genomic Laboratory Hub, Oxford University Hospitals NHS Foundation Trust, Oxford, UK

^42^ Public Health England, London, UK

^43^ Royal Devon and Exeter NHS Foundation Trust, Exeter, UK

^44^ Exeter Genomics Laboratory, Royal Devon and Exeter NHS Foundation Trust, Exeter, UK

^45^ Royal Free London NHS Foundation Trust, London, UK

^46^ Sheffield Children's NHS Foundation Trust, Sheffield, UK

^47^ Department of Clinical Genetics, St George’s University Hospitals NHS Foundation Trust, London, UK

^48^ South East Genomics Laboratory Hub, St George’s University Hospitals NHS Foundation Trust, London, UK

^49^ St James’s Hospital, Dublin, Ireland

^50^ Cancer Genetics Unit, The Royal Marsden NHS Foundation Trust, Sutton, London, UK

^51^ Trinity College Dublin, The University of Dublin, Ireland

^52^ University Hospital of Wales, Cardiff and Vale University Health Board, Cardiff, UK

^53^ University Hospitals Bristol NHS Foundation Trust, Bristol, UK

^54^ University Hospitals of Leicester NHS Trust, Leicester, UK

^55^ University Hospitals of Plymouth NHS Trust, Plymouth, UK

^56^ University of Manchester, Manchester, UK

^57^ Wessex Clinical Genetics Service, Princess Anne Hospital, Southampton, UK

^58^ Faculty of Medicine, University of Southampton, Southampton, UK

^59^ University Hospital Southampton NHS Foundation Trust, Southampton, UK

^60^ Cork University Hospital, Cork, Ireland
